# Protocol for the prevention and management of complications related to ADM implant-based breast reconstructions

**DOI:** 10.3205/iprs000085

**Published:** 2016-01-21

**Authors:** Isabelle Citron, Rory Dower, Mark Ho-Asjoe

**Affiliations:** 1 Department of Plastic Surgery, Guy’s and St Thomas’ Hospital, London, United Kingdom

**Keywords:** Strattice, acellular dermis, breast reconstruction, implant-based, complications, learning curve

## Abstract

Acellular dermal matrix (ADM) implant-based reconstructions have transformed direct-to-implant breast reconstruction (DTI). But like all surgery, it is not deplete of complications such as seroma, infections and wound healing problems. These are cited with varying frequencies in the literature. With increased experience and through a series of measures instituted to minimize complications, we have been able to improve outcomes for our patients. We report our technical refinements for prevention of ADM reconstruction associated complications including patient selection, implant selection, drains, dressing and our post operative antibiotic regime. We also outline our protocol for the management of ADM associated complications including seroma, simple and complex infection and red breast syndrome, such that the sequelae of complications are minimized and patients achieve a better long-term outcome.

## Zusammenfassung

Acelluläre Gewebematrix (ADM) wird routinemäßig bei implantatbasierten Brustrekonstruktionen verwendet. Wie bei allen operativen Eingriffen kann es zu Komplikationen wie z.B. Seromen, Infektionen und einer gestörten Wundheilung kommen. Die Häufigkeitsangaben in der Literatur sind sehr unterschiedlich. Mit zunehmender Erfahrung und einer Reihe von Maßnahmen zur Verringerung der Komplikationsraten konnten die Ergebnisse für unsere Patientinnen verbessert werden. Eine Prävention ADM-assoziierter Komplikationen beinhaltet die Auswahl geeigneter Patientinnen und Implantate, Drainagen und Wundverbände sowie das postoperative Antibiotika-Regime. Darüberhinaus stellen wir unser Protokoll zur Vorgehensweise bei ADM-assoziierten Komplikationen wie Seromen, einfachen und komplexen Infektionen und dem Syndrom der roten Brust vor, mit dem der Schweregrad verringert und die Langzeitergebnisse verbessert werden.

## Introduction

Since the introduction of Strattice™ into the UK in 2009, acellular dermal matrix (ADM) has become a part of the breast reconstruction repertoire at many units. ADM is most commonly used in immediate, single-stage implant breast reconstruction and is gaining popularity in delayed reconstructions [1] and corrections of breast deformities [2]. As an early adopter of the technique, the senior author has used Strattice™ (LifeCell Corp., Branchburg, NJ), a porcine acellular dermal matrix in breast reconstruction for five years. Initial reports of ADM use indicated a high rate of complications especially seroma, infections and wound healing problems [3], [4], [5], [6], [7], [8], [9], [10]. As ADM utilization become more widespread and surgeons have gained more experience, they have iterated and improved their techniques. Accordingly, the rate of complications associated with ADMs has decreased [1].

Although the majority of complications can be easily dealt with, poor management can progress to implant loss. To support and standardize decision making across hospitals and practitioners we have developed a protocol to minimize the occurrence of complications and to ensure complications are appropriately managed so as to minimize the risk of implant loss. This guideline will help shorten the learning curve for units more recently adopting this technique. It is also of use for junior surgeons who may have minimal exposure to ADM breast reconstruction but are often the first clinician to assess these patients when they present with complications.

This paper describes our unit’s approach to patient selection and technical refinements to avoid complications. It defines a protocol for the management of complications to avoid implant loss. Since the introduction of the protocol, we have avoided implant loss over the last two and a half years.

## Patient selection

In our experience, the most important step in reducing the risk of complications when using Strattice™ is patient selection. Factors known to increase the risk of complications associated with the use of ADMs include mastectomy weights >600 g, BMI >30 and smoking [6], [7], [11], [12]. Neoadjuvant chemotherapy whilst not found to be a factor in some studies, showed a trend towards higher complication rates in our unit [13], [14]. Awareness of these risk factors across the MDT allows health professionals to appropriately council high risk patients away from DTI using ADM, towards using an expander as a temporary spacer while leaving the ADM for the second stage in correcting deformities. Patient selection criteria are therefore the most important guideline to follow when trying to minimize complications in ADM-implant based reconstructions.

## Seroma

Seroma is by far the most common complication associated with the use of ADMs. ADM-implant reconstructions, by their very nature, have a number of risk factors known to increase the propensity for seroma formation. These include large surface area wounds following skin sparing mastectomy [15], creation of a large dead space, extensive use of electrocautery [16], patients with high BMI [17], [18] and in certain cases, axillary lymph node clearance [19]. 

We have identified two important factors which minimize seroma formation with ADM implant reconstructions. The first is proper implant selection. A correctly sized implant fills dead space and improves approximation between skin and Strattice™ thus promoting early integration and revascularization. A difficult balance must be struck as larger implants can increase skin flap tension which can compromise the skin flap vascularity and wound edge healing. This is evidenced by an increased complication rate in mastectomies weighing >600 g. Through experience, operators achieve this balance of tight closure without compromising mastectomy flap survival. 

The second important factor in minimizing seroma formation is the use of closed suction drainage. Closed suction drainage allows the removal of seroma fluid, which constitutes a risk factor for infection [20]. It also has a role in shutting down dead space, draining the excess exudate and promoting attachment of the mastectomy flaps to the underlying ADM. Our preference is to place two drains, one between the implant and ADM and one between the mastectomy flaps and ADM. These are allowed to drain for a prolonged period of up to two to three weeks in some cases and are removed when drainage has been recorded as below 30 ml over a 24-hour period.

The disadvantage is that the drain is a conduit to the outside environment and therefore potentially increases the risk of infection to the implant [21]. We utilize a number of strategies to try to minimize this risk including creating a long subcutaneous tunnel when inserting the drain in an attempt to minimize exposure of the implant and Strattice™ to the outside environment. In addition, the drain exit site is kept as clean as possible for the duration of therapy. The use of adjuncts such as bacteriostatic dressings, for example the Biopatch™, used to decrease catheter-related blood steam infections, may be a novel method of reducing drain related infections [22]. 

Despite waiting for drainage to decrease below 30 ml/24 hrs, seromas can still occur after drain removal. In these cases, the seroma should be managed with ultrasound guided drainage under strict aseptic techniques to prevent any super infection of the implant cavity which can threaten the implant.

## Infections and wound healing complications

### Implant exposure

The presence of Strattice™ provides additional layer beneath the skin when there is wound dehiscence or skin necrosis. Exposure of Strattice™ is usually minor at the level of the suture line but occasionally can be more serious when there is necrotic skin involvement.

Minor Strattice™ exposure or eschar over the suture line can occasional be noted in out-patients and in most cases, there is no associated infection. The presence of Strattice™ provides an interface between implant and skin and therefore acts as a barrier to lower the risk of infection. Our protocol advises these wounds have microbiological swabs sent and be dressed with Inadine™ and Mepitel™ and followed up in out-patient clinic. If required, minor surgery for wound closure can be scheduled onto an elective list. 

In most cases of minor Strattice™ exposure, the implant is not affected as the Strattice™ protect the implant. However, if the exposure is associated with a large area of skin necrosis, the implant will need to be removed and exchanged for a deflated expander to assist closure.

### Prevention of infection

Infection is a complication of all surgery, and implant breast reconstruction, with or without Strattice™, is no exception. Weichman et al. demonstrated a 4.76% infection rate in implant reconstruction, the most common organisms being *S. epidermidis*, *S. aureus*, *S. marcescens* and *P. aeruginosa* [8]. 86% were sensitive to gentamycin. There is no consensus as to the ideal duration of antibiotics therapy, with some advocating a single peri-operative dose [23] and others a prolonged course up until drain removal [24]. 

In the author’s experience, the two most common infecting organisms complicating implant breast reconstruction are *S. aureus* and *P. aeruginosa*. Our protocol therefore advocates prevention of infection during the primary procedure through meticulous assessment of mastectomy skin flap vascularity as well as strict sterility. In addition, we advocate soaking the implant and Strattice™ in antibiotic solution (1.2 g amoxicillin/clavulanic acid and 80 mg gentamycin) to minimize infection risk. Peri-operative IV antibiotics (co-amoxiclav or teicoplanin) are also given followed by 5 days oral antibiotics. Routine continuation of antibiotics up until drain removal is not advocated. 

### Management of infection

Despite the best preventative measures, a small number of wound and implant infections still occur. The general management includes a septic screen and appropriate culture directed antibiotic use. This is usually sufficient for mild infections.

More severe implant infections and exposures have traditionally been managed surgically in a 2-stage procedure. The first stage is removal of the affected implant and debridement of non-viable tissue. The second stage, the insertion of a new implant, occurs after a few months once the inflammatory process has subsided. This method commits the patient to a period of asymmetry and loss of the breast skin envelope. The skin envelope requires a prolonged period of expansion before the desired shape is once again obtained and in many cases, additional skin is needed for this type of delayed reconstruction. In many instances, the end result is never as aesthetically pleasing as in a primary reconstruction. A number of techniques have been attempted to circumvent this sub-optimal patient outcome [2].

In patients where there is severe infection, implant removal is inevitable. To avoid converting to a delayed reconstruction in infective cases, our protocol puts forward a new management method with negative pressure dressing. Patients are admitted to hospital and a basic septic screen performed. Empirical IV antibiotics are instituted whilst awaiting culture results. During the operation, the implant is removed and the cavity is washed with copious amount of 50% betadine and hydrogen peroxide. Whether or not the Strattice™ should be removed is decided on a case by case basis. In the author’s experience, non viable, infected or inflamed Strattice™ which requires debridement can be easily identified as it is usually frail and partially hydrolysed. In a method adapted from that originally described by Liao and Breuing [25], a negative pressure dressing sponge is inserted into the resultant cavity. The aim is to fill the cavity to excess and in so doing, mimic the effects of a larger implant. A size 10 FG closed suction drain is then inserted into the cavity and the wound cavity sealed off. The redivac removes the residual fluid from the washout and reduces tissue oedema. However, in our unit, we found prolonged negative pressure deflate the sponge and therefore, unable to maintain the skin envelope. Therefore, we only maintatin negative pressure drainage for overnight and the sponge is allow to re-inflate afterwards.

After 48–72 hours, the patient is again taken to theatre for another washout, new microbiological swab is taken and the sponge replaced. This process is repeated until cultures return a negative finding for any microbial growth. In practice, most patients require a total of two or three visits to theatre until these criteria are met. The patient is then ready for their final procedure: placement of a new implant and final closure of the wound. In some cases where extensive debridement of non viable skin took place, a smaller implant or an expander may then be necessary. However, if the issue was purely infective, this technique allows for retaining the cavity volume and a same size implant can be used.

## Red breast syndrome

Red breast syndrome (RBS) is a non-infectious erythema unique to ADM associated breast reconstructive procedures and ranges between 0–7% [26]. The exact mechanism is yet to be elucidated but RBS was first theorized by Nahabedian [27] with Alloderm who suggested intra-operative washing to remove the causative agent. Several potential causes have been cited in the literature including dependent erythema, altered lymphatic flow, non-specific graft reaction, reaction to chemicals or pharmacologic agents used to process the graft, an unknown histocompatibility factor, generalized inflammatory response, interruption of lymphatic flow and neovascularization of the graft material [27], [28], [29]. Clinical presentation is that of erythema over the area where the Strattice™ has been inset. Patients typically do not complain of pain and the breast is not hot to touch which differentiates this from cellulitis or acute infection. In the majority of cases, the redness is localised to the infero-lateral aspect of the breast where the Strattice™ is used for the reconstruction in the early stage. To manage these cases, our guideline suggests assessment of inflammatory markers including C-reactive protein (CRP) and white cell count. The white cell is usually within the normal range and the CRP is usually mildly elevated and around the range between 50–100 mg/L. If these are not disproportionately elevated then patients are reassured and followed up only if symptoms worsen. Procalcitonin levels is an area of ongoing research [26]. However no test is is sufficiently specific to give a definitive result and a diagnosis of RBS must be based on clinical experience. Although antibiotics are not indicated [30] the use of anti-histamine and corticosteroids is debatable [26]. In one case at our institution, one patient who developed gross dermatitis with RBS had a positive response to these agents . Our current policy is to keep patient under close observation and prescribe NSAIDs unless there is significant symptoms and signs for infection.

## Summary

The use of Strattice™ has transformed the senior author’s practice in breast reconstruction over the last four years. It has become the main alternative to free autologous tissue transfer and overtaken the use of Latissimus dorsi in primary reconstruction.

With implant reconstruction, the major fear is the need to remove the implant resulting in skin contraction leading to a delayed reconstruction with poor aesthetic outcome. Having performed over 100 cases using Strattice™ for breast reconstruction at our unit, we have learnt from our experience and reduced the occurrence of complications. However a low level of complications still exists. Using the protocol set out in this paper to manage these complications, for the past two and a half years, early complications have been appropriately managed such that not a single implant has been lost. The protocol is now in use as a guideline to avoid implant loss and allow the widespread use of Strattice™ in a one stage immediate reconstruction. We would encourage the implementation of this protocol in other units.

## Notes

### Competing interests

M. Ho-Asjoe is a speaker for Lifecell.
